# Meiotic chromosome axis remodelling is critical for meiotic recombination in *Brassica rapa*

**DOI:** 10.1093/jxb/erab035

**Published:** 2021-01-27

**Authors:** Maria Cuacos, Christophe Lambing, Miguel Pachon-Penalba, Kim Osman, Susan J Armstrong, Ian R Henderson, Eugenio Sanchez-Moran, F Christopher H Franklin, Stefan Heckmann

**Affiliations:** 1 Leibniz Institute of Plant Genetics and Crop Plant Research (IPK) OT Gatersleben, D-06466 Seeland, Germany; 2 Department of Plant Sciences, University of Cambridge, Cambridge CB2 3EA, UK; 3 School of Biosciences, University of Birmingham, Edgbaston, Birmingham B15 2TT, UK; 4 Ohio State University, USA

**Keywords:** ASY1, *Brassica rapa*, crossover, meiosis, meiotic chromosome axis remodelling, meiotic recombination, PCH2, synaptonemal complex

## Abstract

Meiosis generates genetic variation through homologous recombination (HR) that is harnessed during breeding. HR occurs in the context of meiotic chromosome axes and the synaptonemal complex. To study the role of axis remodelling in crossover (CO) formation in a crop species, we characterized mutants of the axis-associated protein ASY1 and the axis-remodelling protein PCH2 in *Brassica rapa*. *asy1* plants form meiotic chromosome axes that fail to synapse. CO formation is almost abolished, and residual chiasmata are proportionally enriched in terminal chromosome regions, particularly in the nucleolar organizing region (NOR)-carrying chromosome arm. *pch2* plants show impaired ASY1 loading and remodelling, consequently achieving only partial synapsis, which leads to reduced CO formation and loss of the obligatory CO. PCH2-independent chiasmata are proportionally enriched towards distal chromosome regions. Similarly, in Arabidopsis *pch2*, COs are increased towards telomeric regions at the expense of (peri-) centromeric COs compared with the wild type. Taken together, in *B. rapa*, axis formation and remodelling are critical for meiotic fidelity including synapsis and CO formation, and in *asy1* and *pch2* CO distributions are altered. While *asy1* plants are sterile, *pch2* plants are semi-sterile and thus *PCH2* could be an interesting target for breeding programmes.

## Teaser

ASY1 and PCH2 are critical for meiotic axis remodelling as well as synaptonemal complex and crossover formation in *Brassica rapa*. Residual crossovers in *asy1* and *pch2* plants show altered patterning.

## Introduction

Meiosis is a specialized cell division in sexually reproducing organisms shuffling maternal and paternal genomes through homologous recombination (HR) and independent assortment of homologous chromosomes.

HR is initiated by programmed formation of SPO11-catalysed DNA double-strand breaks (DSBs) (Keeney and Kleckner, 1995; Keeney *et al.*, 1997; Neale *et al.*, 2005; Pan *et al.*, 2011). DSBs undergo a series of transitions, being repaired differently (Osman *et al.*, 2011; Mercier *et al.*, 2015; Wang and Copenhaver, 2018) into either a crossover (CO; reciprocal genetic exchange between homologous chromosomes) or a non-crossover (NCO), using as repair template either the sister chromatid or the homologous chromosome exchanging only short stretches of DNA. Erroneous meiotic DSB formation or repair may lead to univalent formation or chromosome breakage and thus genome instability, reducing fertility. In plants <10% of DSBs are repaired as COs, and DSBs/COs occur heterogeneously along chromosomes (Muyt *et al.*, 2009; Mercier *et al.*, 2015) limiting genetic variation generated during each meiotic cycle. In many crop species, COs are restricted to chromosome ends, inhibiting access to traits residing in ‘cold’ regions or creating ‘linkage drag’; therefore, modulating CO number and distribution is of interest for plant breeding (Phillips *et al.*, 2013; Choulet *et al.*, 2014; Li *et al.*, 2015; Demirci *et al.*, 2017; Lambing and Heckmann, 2018; Dreissig *et al.*, 2019).

CO formation is tightly controlled in the following ways: (i) interhomologue bias, where recombination is favoured between homologous chromosomes; (ii) CO assurance, where at least one ‘obligate’ CO forms per chromosome essential for faithful chromosome segregation; and (iii) CO interference, where formation of one CO reduces the probability of another close by, thereby hampering CO clustering and limiting CO number per chromosome (Zickler and Kleckner, 2015). In Arabidopsis, at least two CO classes exist: ~85% of COs are catalysed by the ZMM proteins (MER3, HEI10, ZIP4, SHOC1, PTD, MSH4, and MSH5) and MLH1–MLH3 (Higgins *et al*., 2004, 2008*b*; Chen *et al.*, 2005; Mercier *et al.*, 2005; Wijeratne *et al.*, 2006; Chelysheva *et al*., 2007, 2012; Macaisne *et al.*, 2008), generating interference-sensitive class I COs; and part of the remaining ~15% are MUS81- or FANCD2-dependent interference-insensitive class II COs (Berchowitz *et al.*, 2007; Higgins *et al.*, 2008a; Kurzbauer *et al.*, 2018).

Concurrently with HR, the meiotic nucleus undergoes extensive reorganization of the chromatin. Following S phase, sister chromatids are linked by cohesins and, during leptotene, are organized in chromatin loops tethered to a linear proteinaceous structure called the meiotic chromosome axis (Kleckner, 2006). In Arabidopsis, this structure comprises, for example, ASY1, ASY3, ASY4, SMC3, and REC8 (Caryl *et al.*, 2000; Cai *et al.*, 2003; Chelysheva *et al.*, 2005; Lam *et al.*, 2005; Ferdous *et al.*, 2012; Chambon *et al.*, 2018). DSBs form at preferred sites (hotspots) in the chromatin loops which are then tethered to the axes where they are repaired as COs or NCOs (Panizza *et al.*, 2011). Following DSB formation, a proteinaceous structure, called the synaptonemal complex (SC), starts to form during zygotene by progressively polymerizing between homologous chromosome axes physically connecting them and promoting CO formation (Page and Hawley, 2004).

The Arabidopsis meiotic axis-associated protein ASY1 (Caryl *et al.*, 2000; Armstrong *et al.*, 2002; Sanchez-Moran *et al.*, 2007) is a HORMA domain-containing protein (yeast HOP1, mouse HORMAD1/2, or rice PAIR2; Hollingsworth *et al.*, 1990; Caryl *et al.*, 2000; Nonomura *et al.*, 2004; Fukuda *et al.*, 2010) required for synapsis and HR. In different organisms it is involved in the interhomologue bias (Martinez-Perez and Villeneuve, 2005; Niu *et al.*, 2005; Sanchez-Moran *et al.*, 2007; Carballo *et al.*, 2008; Kim *et al.*, 2010). In plants, *asy1* or *pair2* mutants display asynapsis and univalents due to reduced chiasma formation (Ross *et al.*, 1997; Caryl *et al.*, 2000; Nonomura *et al.*, 2004). In addition to axis and SC formation *per se*, dynamic regulation of these structures is critical for CO formation. Concomitant with ZYP1 loading, ASY1 becomes depleted from synapsed regions in a PCH2-dependent manner (Wojtasz *et al.*, 2009; Chen *et al.*, 2014; Lambing *et al.*, 2015). PCH2 is a conserved AAA-ATPase with diverse functions in different organisms. Initially reported as a checkpoint protein in yeast (San-Segundo and Roeder, 1999) and other species (Bhalla and Dernburg, 2005; Joyce and McKim, 2009), it has also been implicated in numerous meiotic processes, including DSB/CO formation, interhomologue bias, interference, axis morphogenesis, synapsis, and inhibition of DSB formation at rDNA borders (Börner *et al.*, 2008; Joshi *et al.*, 2009., 2015; Wojtasz *et al.*, 2009; Zanders and Alani, 2009; Vader *et al.*, 2011; Zanders *et al.*, 2011; Farmer *et al.*, 2012; Miao *et al.*, 2013; Lambing *et al.*, 2015; Subramanian *et al.*, 2016). In plants, Arabidopsis PCH2 is critical for axis remodelling as well as SC and CO formation (Lambing *et al.*, 2015; Yang *et al*., 2020a, b) while rice PCH2 (CRC) is an integral component of the SC essential for DSB and CO formation as well as ASY1 assembly (Miao *et al.*, 2013). Interestingly, in Arabidopsis *pch2*, CO rates are altered in some chromosome regions (Lambing *et al.*, 2015).

To study the role of axis remodelling in a crop species, we characterized *asy1* and *pch2* mutants in *Brassica rapa* (2*n*=20). *asy1* plants form axes but SC formation is defective, leading to a reduction in CO frequency. Interestingly, residual chiasmata are proportionally increased towards chromosome ends and are enriched in the major 45S rDNA-carrying chromosome; in particular, chiasmata form within or close to the 45S rDNA locus while this is not the case in the wild type (WT) or in *pch2*. *pch2* plants form a partial SC while axes are not remodelled; that is, ASY1 loading during leptotene is reduced and ASY1 is not depleted from synapsed regions, leading to a reduction in CO frequency. PCH2-independent chiasmata are more skewed towards terminal chromosome regions. To corroborate this cytological observation, in Arabidopsis CO rates were established in different genetic intervals, revealing increased CO rates in telomeric chromosome regions at the expense of decreased (peri-)centromeric CO rates in *pch2* compared with the WT. We conclude that ASY1 and PCH2 are critical for SC and CO formation during meiosis in *B. rapa* and that *asy1* and *pch2* show altered CO patterning. Due to *pch2* showing only semi-sterility, it could potentially be an interesting target in breeding programmes to redistribute COs.

## Materials and methods

### Plant material

*Brassica rapa* cultivar R-o-18 and Arabidopsis ecotype Columbia (Col-0) were used as the WTs. The following *B. rapa* mutant lines were received from RevGenUK (https://www.jic.ac.uk/research-impact/technology-research-platforms/reverse-genetics/): *asy1-13* (JI32391-B), *asy1-14* (JI31044-B), *pch2-9* (JI32373-B), and *pch2-12* (JI32174-B). The following Arabidopsis T-DNA insertion lines (Alonso *et al.*, 2003) in the Col-0 background were obtained from the T-DNA mutant collection at the Salk Institute Genomics Analysis Laboratory (SIGnAL, http://signal.salk.edu/cgi-bin/tdnaexpress) via the NASC (http://arabidopsis.info/): *asy1-4* (SALK_046272) (Lambing *et al.*, 2020), *shoc1-1* (SALK_057589) (Macaisne *et al.*, 2008), *mus81-2* (SALK_107515) (Higgins *et al.*, 2008a), and *pch2-1* (Sail_1187_C06) (Lambing *et al.*, 2015). Plants were grown in greenhouses under 16 h day/8 h night, at 16 °C/14 °C (*B. rapa*) and 21 °C/18 °C (Arabidopsis) day/night temperatures.

### Genotyping

In *B. rapa*, PCR-based genotyping was performed using derived cleaved amplified polymorphic sequences (dCAPS) (Neff *et al.*, 1998) with primers selected using the dCAPS Finder 2.0 tool (http://helix.wustl.edu/dcaps/) (Neff *et al.*, 2002). A list of primers and restriction enzymes is shown in Supplementary Table S1. After PCR amplification, the resulting amplicons were digested with corresponding restriction enzymes at 37 °C overnight and resolved on 2.5% agarose gels. Primers used to genotype Arabidopsis T-DNA mutants are indicated in Supplementary Table S1.

### RNA extraction and reverse transcription–PCR

Total RNA of sample tissues was extracted with an RNeasy Plant Mini Kit (Qiagen) performing DNase digestion with the RNase-Free DNase Set (Qiagen). Reverse transcription was performed in a 20 µl reaction employing 750 ng of total RNA with a Tetro cDNA synthesis kit (Bioline) using oligo(dT) primers. Expression of *Actin* was evaluated as technical control for the integrity of the RNA/cDNA. A 5 µl aliquot of undiluted cDNA was used as template for 36 (*PCH2-12*) and 28 (*Actin*) PCR cycles. Primers used are listed in Supplementary Table S1.

### Cytology and microscopy

Cytological procedures were carried out as described (Armstrong *et al.*, 2009) with minor modifications. 5S (pCT4.2; Campell *et al.*, 1992) and 45S (pTa71; Gerlach and Bedbrook, 1979) rDNA fluorescence *in situ* hybridization (FISH) probes were labelled by nick translation with Texas Red and Atto488 (NT labelling kits, Jena Biosciences). Chromosome spreading for immunostaining in *B. rapa* was done using five anthers per slide; digestion was done during 8 min in a moist chamber at 37 °C, disrupting the material with a brass rod after the first 4 min; and spreading was done with 1.5% lipsol. Immunostaining in Arabidopsis was performed on acid chromosome spreads from male meiocytes as described (Lambing *et al.*, 2020). The following antibodies and dilutions were used: anti-ASY1 (rabbit/rat, Sanchez-Moran *et al.*, 2007, 1:500), anti-ZYP1 (rat/guinea pig, Higgins *et al.*, 2005, 1:500), anti-SMC3 (rat, Ferdous *et al.*, 2012, 1:300), anti-ASY3 (rabbit, Ferdous *et al.*, 2012, 1:200), anti-MLH1 (for *B. rapa* rabbit, Jackson *et al.*, 2006; for Arabidopsis rabbit, Chelysheva *et al.*, 2010, 1:200), and anti-HEI10 (rat, Lambing *et al.*, 2015, 1:200). ASY1 intensity in *B. rapa* asynapsed versus synapsed regions was measured as described (Lambing *et al.*, 2015). Quantification of ASY1 signal intensity in Arabidopsis leptotene nuclei was performed according to Ziolkowski *et al.* (2017). In brief, Arabidopsis images were acquired as *z*-stacks of 10 images with an optical section of 0.2 µm each. The maximum fluorescence intensity projection for each cell was generated, and total signal intensity was quantified using ImageJ. A region adjacent to the chromatin was used to quantify the background level that was deducted from the total signal intensity. Each signal intensity was normalized by the mean WT signal intensity for comparison. Quantification of ASY1 signal intensity in *B. rapa* leptotene nuclei was performed in a similar way, but images were acquired as a single plane, and measurements were done with the corresponding microscope software. Images were acquired with a Nikon Eclipse 90i fluorescence microscope equipped with a Nikon DS-Qi1Mc digital camera and NIS-Elements-F software, or a Nikon Eclipse Ni-E equipped with a Nikon DS-Qi2 camera and NIS-Elements-AR version 4.60 software (Nikon, Tokyo, Japan). Images were processed with GIMP 2.10 (www.gimp.org).

### Recombination measurement at genetic intervals

Recombination measurements for the intervals I5b, I2f, I2g, I5a, I5d, and I5c using fluorescent pollen (Berchowitz and Copenhaver, 2008) are from the study of Lambing *et al.* (2015); 420 and 5.11 are seed-based fluorescent assays (Melamed-Bessudo *et al.*, 2005; Wu *et al.*, 2015). Fluorescent seeds and recombination measurements were calculated using Cell Profiler as previously described (Ziolkowski *et al.*, 2015). CEN3 is a pollen-based fluorescent assay (Berchowitz and Copenhaver, 2008; Yelina *et al.*, 2012). Fluorescent pollen was manually recorded under an epifluorescence microscope. and recombination measurements were calculated as previously reported (Lambing *et al.*, 2015).

## Results

### Isolation of *Brassica rapa asy1* and *pch2*

The *B. rapa* genome encodes *ASY1* (Bra004222) and *PCH2* (Bra013827) on chromosomes A07 and A01, respectively. One region spanning ~1 kb within *ASY1* and within *PCH2* were screened in a *B. rapa* R-o-18 TILLING (targeting induced local lesions in genomes) population (Stephenson *et al.*, 2010) by the RevGenUK TILLING service (https://www.jic.ac.uk/research-impact/technology-research-platforms/reverse-genetics/) to isolate putative *asy1* and *pch2* loss-of-function alleles.

**Fig. 1.**
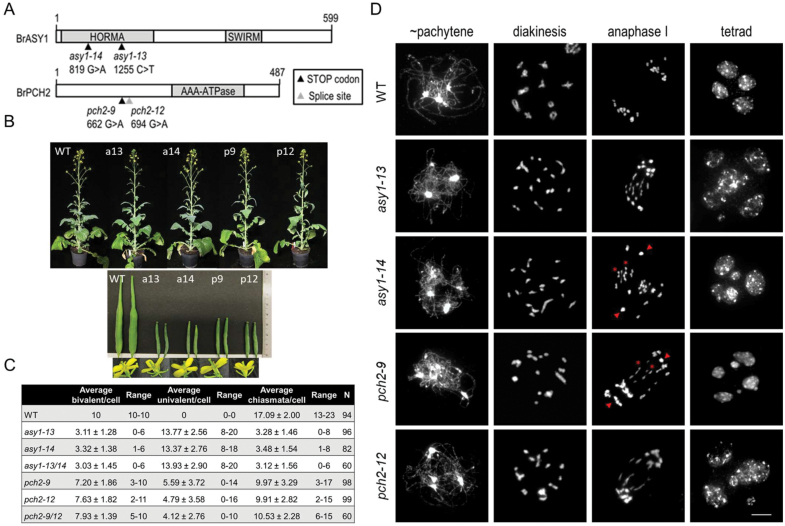
*asy1* and *pch2* plants display reduced fertility due to defects during meiosis. (A) Schematic representation of *Brassica rapa* ASY1 and PCH2 including mutations analysed (arrowheads). (B) *B. rapa* plant phenotypes (upper panel) including siliques and flowers (lower panel): WT, *asy1-13* (a13), *asy1-14* (a14), *pch2-9* (p9), and *pch2-12* (p12). (C) Average, SD, and range of bivalents, univalents, and chiasmata per cell in analysed lines. *n*=number of cells; a minimum of two independent plants per genotype. (D) *B. rapa* WT, *asy1*, and *pch2* male meiotic chromosome spreads. In both mutants, synapsis and chiasma formation are impaired, leading to the occurrence of univalents at diakinesis that results in unequal chromosome segregation and unbalanced tetrads. Note, univalents in the mutants could migrate complete to a pole (arrowheads) or separate chromatids (asterisks) during anaphase I. DNA is counterstained with DAPI and shown in grey. Scale bar=10µm.

For *ASY1*, two lines with predicted premature stop codons were selected (Fig. 1A): *asy1-13* (1255C>T, Q143>Stop) and *asy1-14* (819G>A, W72>Stop) predicted to encode 24% and 12% of the native protein length, respectively. Sequencing analysis in each line confirmed the presence of a single nucleotide polymorphism (SNP).

For *PCH2*, one line with a predicted premature stop codon and a second line with an SNP in an intron–exon boundary were selected (Fig. 1A): *pch2-9* (662G>A, W143>Stop), predicted to encode 29% of the native protein length, and *pch2-12* (694G>A, intron4–exon4 boundary). SNPs were confirmed by sequencing. Reverse transcription–PCR revealed the presence of alternative splice variants in *pch2-12* (Supplementary Fig. S1). Although the presence of the *PCH2* WT transcript cannot be excluded, *pch2-12* probably results in a null mutation as *pch2-12* is allelic to *pch2-9* in terms of meiotic fidelity (see below). *PCH2* expression is found in flower buds (containing cells undergoing meiosis) and also in leaves, similar to the expression pattern found for other meiotic genes (Caryl *et al.*, 2000; Grelon *et al.*, 2001).

For all lines, three backcrosses to the WT (R-o-18) were performed in order to decrease the secondary mutation load. Data presented correspond to plants from the third backcross generation.

### *asy1* and *pch2* plants have reduced fertility due to defects in meiosis

All plants exhibited normal vegetative growth and development (Fig. 1B). However, plants homozygous for any of the SNPs showed sterile anthers, short siliques, and reduced seed set, while corresponding heterozygous or WT TILLING plants were similar to the WT (Fig. 1B; Supplementary Table S2). To explore whether reduced fertility was due to meiotic defects, we performed male meiotic chromosome spread analysis. During WT meiosis (Fig. 1D; Supplementary Fig. S2a), unpaired chromosomes appear as thin threads during leptotene. During zygotene, chromosomes start to align and synapse, reaching full synapsis visible as thick threads at pachytene. At diakinesis, homologous chromosomes are visible as bivalents physically connected by chiasmata (cytological manifestation of COs). At metaphase I, 10 bivalents align at the equatorial plate and 10 homologous chromosomes migrate to opposite poles during anaphase I. During the second meiotic division, chromosomes align at metaphase II and 10 chromatids separate to opposite poles during anaphase II, producing four haploid products in a balanced tetrad.

In *asy1*, thick chromatin threads indicative of synapsis were not observed (Fig. 1D). During diakinesis and metaphase I, most chromosomes appeared as univalents, indicating a failure to form COs. During anaphase I, univalents either segregated to one pole or showed precocious separation of sister chromatids (Fig. 1D), leading to unbalanced tetrads and micronuclei. The WT showed invariably 10 bivalents with a mean of 17.1±2 chiasmata per cell, while *asy1* showed univalents in all cells. Reduced chiasma values for both *asy1* lines (3.28±1.46 and 3.48±1.54 chiasmata per cell for *asy1-13* and *asy1-14*, respectively) were not significantly different (Student’s *t-*test, *P*=0.39, *n*=96 *asy1-13* and *n*=82 *asy1-14*) (Fig. 1C). Typically, bivalents formed rods (one chiasma). Only 12.4% of cells were found with one ring bivalent (at least one chiasma on each chromosome arm) and just 2.2% of cells had two ring bivalents (22/178 and 4/178 cells, respectively). No difference was found in *asy1-13/14* compared with each single mutant, confirming that the mutations are allelic (Fig. 1C; Supplementary Fig. S2c; Supplementary Table S2). Moreover, heterozygous *ASY1* plants pollinated with WT pollen produced long siliques with WT seed levels, while WT-pollinated homozygous *asy1* plants produced short siliques with zero seeds, suggesting that female meiosis is also defective. TILLING plants, WT or heterozygous for the mutations, showed WT-like meiosis (Supplementary Fig. S2b). Together, reduced fertility in *asy1* is based on defective synapsis and CO formation, and unbalanced chromosome segregation.

In *pch2* plants, cells with complete synapsis were never observed. At diakinesis/metaphase I, 89% of cells showed a mixture of bivalents and univalents (0–16 univalents per cell) that led to unbalanced tetrads and micronuclei (Fig. 1D). Univalents during anaphase I either separated chromatids or migrated to one pole (Fig. 1D). WT or heterozygous TILLING plants for *PCH2* showed WT-like meiosis (Supplementary Fig. S2b). The mean bivalent number was 7.20±1.86 (range 3–10) with an average of 9.97±3.29 (range 3–17) chiasmata per cell in *pch2-9*, and 7.63±1.82 (range 2–11) bivalents with an average of 9.91±2.82 (range 2–15) chiasmata per cell in *pch2-12* (Fig. 1C). Chiasma values for both lines were not significantly different (Student’s *t*-test, *P*=0.89, *n*=98 *pch2-9* and *n*=99 *pch2-12*). Similar to *asy1*, ring bivalents co-existed with univalents. Notably, 46% of all cells showed ≥10 chiasmata per cell (a number that would be sufficient for all chromosome pairs to acquire at least one obligate CO), together with univalents. An allelism test crossing revealed no difference between *pch2-9/12* and *pch2-9* or *pch2-12* in terms of chiasma formation (Fig. 1C; Supplementary Fig. S2c), supporting that both mutations are allelic. However, in *pch2-12*, despite three backcrosses, a slight reduction in fertility was found among segregating families independent of *PCH2* (Supplementary Table S2). Thus, we focused our analysis on *pch2-9*.

### Increased chiasmata in the major 45S rDNA locus-carrying chromosome in *asy1*

**Fig. 2.**
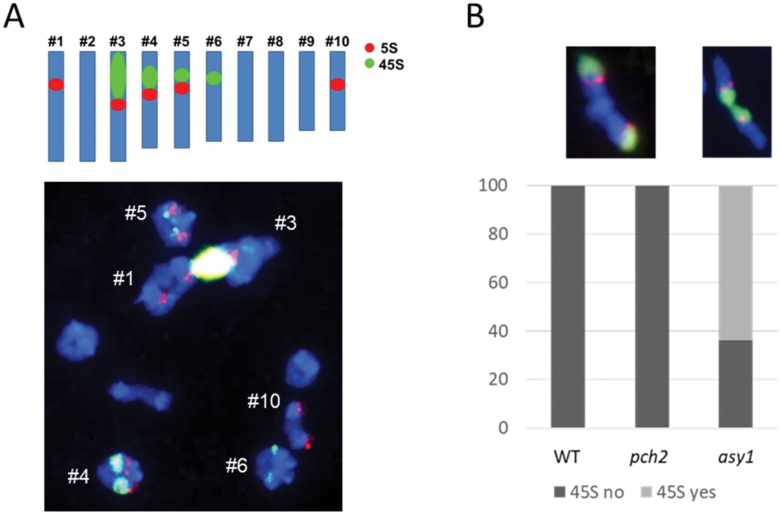
The major 45S rDNA locus-carrying chromosome arm shows increased chiasmata in *asy1* but not in *pch2* or the WT. (A) Top: *B. rapa* chromosomes organized by size with 5S and 45S rDNA clusters detected by FISH. Bottom: a representative WT cell after FISH. (B) Percentage of #3 rod bivalents with a chiasma in the 45S-carrying arm (‘45S yes’, example FISH image right) or in the opposite arm (‘45S no’, example FISH image left). Total number of rod bivalents scored: WT 41, *pch2* 16, *asy1* 36. FISH images: 5S (red), 45S (green); DNA is counterstained with DAPI and shown in blue.

To identify whether in *asy1* bivalents formed preferentially between any particular chromosome pair(s), we performed FISH with 5S and 45S rDNA probes (Fig. 2). The number of 5S and 45S signals in *B. rapa* differs among varieties (Fukui *et al.*, 1998; Snowdon *et al.*, 2002; Koo *et al.*, 2004; Lim *et al.*, 2005; Mun *et al.*, 2010; Xiong and Pires, 2011; Perumal *et al.*, 2017) and has already been discussed (e.g. Hasterok *et al.*, 2006; Boutte *et al.*, 2020). In *B. rapa* R-o-18, we found four 45S and five 5S FISH signals, and assigned them to the chromosomes organized from the largest to the smallest, thereby distinguishing chromosome pairs #1 (largest chromosome, carrying 5S signals), #3 [large block of 45S, the nucleolar organizing region (NOR), and small 5S, probably corresponding to chromosome A03], #4 and #5 (both containing 5S and 45S, with a larger 45S locus in #4), #6 (45S only), and #10 (smallest chromosome, 5S only) (Fig. 2A).

In *asy1*, except for #3, all discernible chromosomes including the largest and the smallest appeared as univalents at similar frequencies (79–86%) (Supplementary Table S3). However, #3 which possesses the NOR with the largest 45S block, appeared as univalent in only 37% of cells. From the 63% #3 bivalents, 84% were rods and 16% rings; and from the #3 rod bivalents, in 64% of *asy1* cells the chiasma was cytologically associated with the 45S rDNA; that is, the chiasma was found either inside the NOR or distal to it (Fig. 2B). In contrast to *asy1*, in WT and *pch2* #3 rod bivalents, the single chiasma was never associated with the NOR but instead was invariably formed in the opposite arm (Fig. 2B).

### Chromosome axes form in *asy1* but fail to assemble a synaptonemal complex

**Fig. 3.**
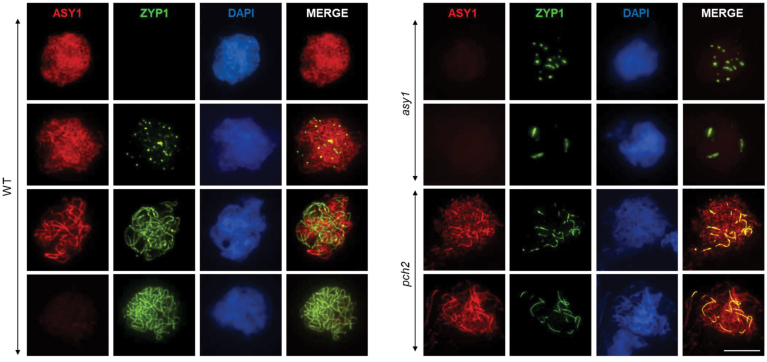
Synapsis is defective in *asy1* and *pch2*. Immunolocalization of ASY1 (red) and ZYP1 (green) in the WT, *asy1*, and *pch2*. In *asy1*, ASY1 is not detected and ZYP1 forms foci in zygotene–pachytene cells and aggregates in late pachytene–diplotene cells. In *pch2*, different from the WT, ASY1 does not get depleted from the axes and appears highly abundant in limited synapsed regions co-localizing with ZYP1. DNA is counterstained with DAPI and shown in blue. Scale bar=10µm.

The *asy1* mutants encode <25% of the native protein length, thus having a truncated or deleted HORMA domain and closure motif, critical for ASY1 localization (West *et al.*, 2019; Yang *et al.*, 2020b). To determine if the truncated ASY1 protein localizes to the axes and an SC forms in the *asy1* cells, we performed ASY1 and ZYP1 (transverse filament protein of the SC; Higgins *et al.*, 2005) immunolocalization. In the WT (Fig. 3), ASY1 localized to chromosome axes during leptotene. Once ASY1 was fully polymerized, ZYP1 initially formed foci and then short stretches which progressively elongated until all homologous chromosomes were fully synapsed. In *asy1* (Fig. 3), ASY1 could not be detected. ZYP1 predominantly formed foci (from <5 to >20, possibly in a stage-dependent manner) and rarely elongated beyond forming short stretches, indicating that SC formation is largely impaired. In late pachytene-/early diplotene-like cells, ZYP1 formed aggregates probably due to its inability to assemble the SC (Zickler and Kleckner, 1999).

**Fig. 4.**
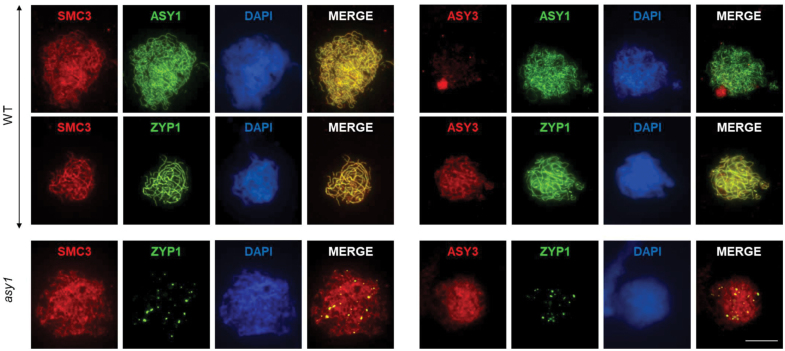
ASY3 and SMC3 localize to chromosome axes in *asy1.* Immunolocalization of SMC3 (red, left) and ASY3 (red, right) together with ASY1 (green, WT top) and ZYP1 (green, WT bottom and *asy1*). ASY3 and SMC3 display similar dynamics in WT and *asy1* cells. DNA is counterstained with DAPI and shown in blue. Scale bar=10µm.

To analyse axis morphogenesis in *asy1*, we performed immunolocalization of the cohesin subunit SMC3 (Lam *et al.*, 2005) and the coiled-coil protein ASY3 (Ferdous *et al.*, 2012). In the WT (Fig. 4), SMC3 was present from leptotene to pachytene, co-localizing initially with ASY1 and later on with ZYP1. Upon synapsis, ASY1 signals became faint while SMC3 persisted, gradually thickening as synapsis proceeded. ASY3 initially localized to the nucleolus during leptotene similar to Arabidopsis (Ferdous *et al.*, 2012) before it co-localized with ASY1 and then with ZYP1; however, ASY3 signals were patchier and less linear than those of ZYP1. In *asy1* (Fig. 4), both SMC3 and ASY3 localized to the axis following WT dynamics. Thus, ASY1-independent axes form but are insufficient to support SC formation, resulting in reduced CO formation.

### Class I and class II crossovers are reduced in *asy1*

**Fig. 5.**
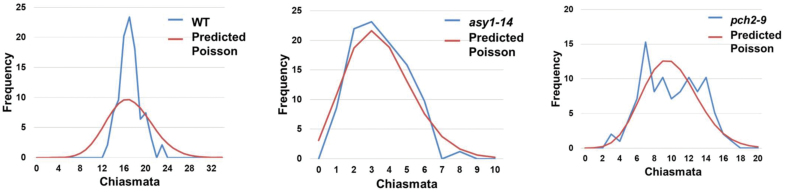
Chiasma frequency distribution follows a Poisson distribution in *asy1* and *pch2*. Chiasma frequency distribution (blue) and predicted Poisson distribution (red) for the WT, *asy1-14*, and *pch2-9*. In the mutants, chiasma frequency distribution does not significantly deviate from a Poisson-predicted distribution.

CO formation was reduced but not abolished in *asy1*. From 178 *asy1* cells analysed, only one cell showed 20 univalents, whereas in all others between one and eight chiasmata were observed. To evaluate the nature of the remaining COs in *asy1*, we analysed the chiasma frequency distribution per cell. In the WT, the majority of COs are sensitive to interference, which leads to a non-random numerical distribution between cells. As a result, the mean chiasma frequency significantly deviates from a Poisson distribution [χ ^2^_(R-o-18)_=46.32, *P*<0.0001, *n*=94] (Fig. 5). However, in *asy1*, the chiasma frequency per cell was not significantly different from a Poisson distribution [χ ^2^_(*asy1-14*)_=7.79, *P*=0.25, *n*=82] (Fig. 5), suggesting that residual ASY1-independent COs are randomly distributed between cells.

**Fig. 6.**
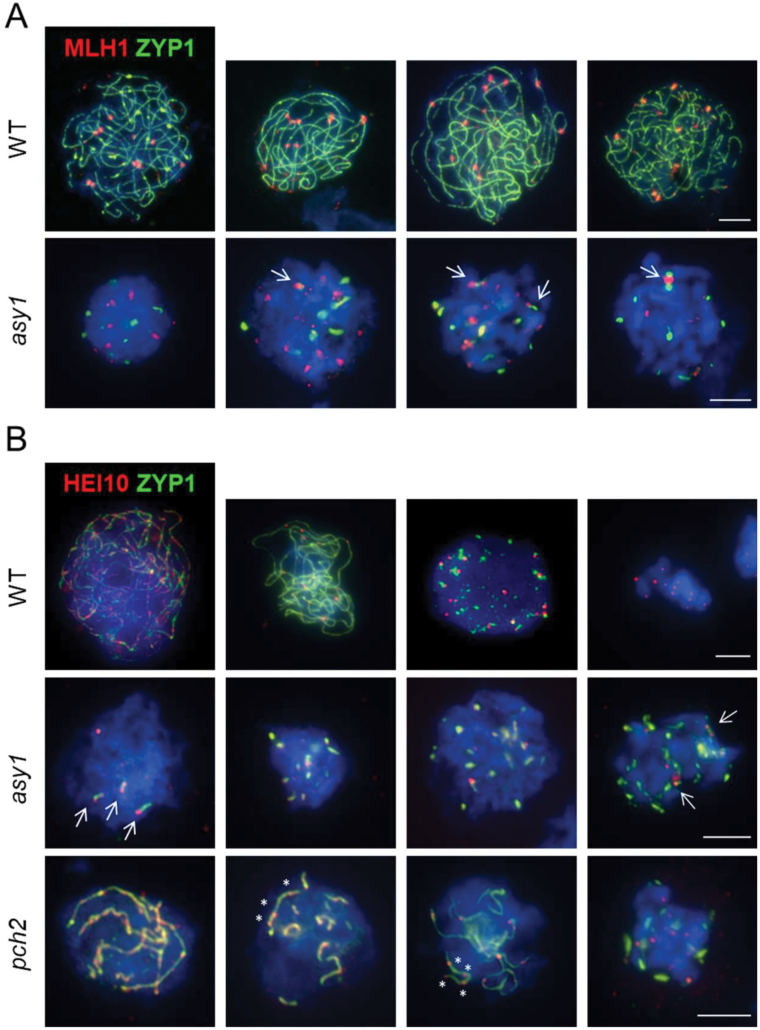
Class I CO formation in *asy1* and *pch2*. Immunolocalization of MLH1 (A, red) and HEI10 (B, red) together with ZYP1 (green) in the WT, *asy1*, and *pch2*. In *asy1*, a subset of MLH1/HEI10 foci co-localize with ZYP1 foci/short stretches (arrows). In *pch2*, several HEI10 signals appear close to each other in a single ZYP1 stretch (asterisks). Note, to depict HEI10 dynamics in the WT, cells are shown ranging from zygotene (left) to diakinesis (right), whereas in all other cases representative examples of cells used for quantification are shown. DNA is counterstained with DAPI and shown in blue. Scale bars=5µm.

To better understand CO formation in *asy1*, we performed immunolocalization of the class I CO marker MLH1 (Jackson *et al.*, 2006) together with ZYP1. We scored on average 9.44±0.72 (*n*=37) and 8.7±4.79 (*n*=10) MLH1 foci per cell in the WT and *asy1*, respectively (Fig. 6A). The MLH1 foci number per cell was highly variable in *asy1* (1–15) but not in the WT (8–11). While in the WT MLH1 foci typically overlapped with ZYP1, in *asy1* only an average of 3.9 MLH1 foci co-localized with residual ZYP1 foci (range 0–9). This manifested in different ways: ZYP1 stretches appeared to originate from MLH1 foci, two MLH1 foci were ‘bridging’ a ZYP1 stretch, or an MLH1 focus formed between two ZYP1 foci (Fig. 6A). Assuming that only ZYP1-associated MLH1 foci or a subset of these are CO competent, at least some of the residual chiasmata in *asy1* could be ZMM-dependent class I COs.

We also immunolocalized HEI10 (Chelysheva *et al.*, 2012; Wang *et al.*, 2012) together with ZYP1. In the WT, numerous small HEI10 foci found during early pachytene progressively fade, leaving on average 11.31±1.71 bright HEI10 foci per cell (*n*=27, range 8–15) during diplotene–diakinesis (Fig. 6B). In *asy1*, this number was significantly lower and more variable (9±3.3, *n*=19, range 4–14). This variability could be attributed to the difficulty in cytologically defining meiotic prophase stages in *asy1* and it is possible that some of the observed HEI10 foci will not mature in CO sites. However, as in the WT, in *asy1*, HEI10 foci typically co-localized with ZYP1. Similar to MLH1, short ZYP1 stretches originated from HEI10 foci or two HEI10 foci bridged a ZYP1 stretch. Altogether, our data suggest that interference-sensitive COs are designated in *asy1*, but the synaptic defect compromises CO maturation. As a result, an obligate CO fails to form between all chromosome pairs, and those COs that do mature exhibit a random distribution.

Next, we asked whether class II COs also form in the absence of ASY1 by taking advantage of available Arabidopsis resources. We crossed Arabidopsis *asy1* either with *shoc1* (involved in class I CO formation; Macaisne *et al*., 2008, 2011) or with *mus81* (involved in class II CO formation; Berchowitz *et al.*, 2007; Higgins *et al.*, 2008a), and compared the chiasma frequency in these lines. Under our growth conditions, Arabidopsis *asy1* showed 2.3±0.88 bivalents and 2.77±1.22 chiasmata per cell (Table 1), *shoc1* showed 2.38±1.04 bivalents and 2.79±1.41 chiasmata per cell, and *mus81* invariably showed five bivalents. In *asy1/shoc1* and in *asy1/mus81*, fewer chiasmata formed than in *asy1*; in *asy1/shoc1*, we found a reduction of ~72% and in *asy1/mus81* a reduction of ~21% compared with *asy1*. This suggests that residual COs in *asy1* are both class I and II.

**Table 1.** ASY1-independent CO formation in *Arabidopsis thaliana*

	Average bivalent/cell	Range	Average chiasmata/cell	Range	Average ring/cell	Range	*n*
*asy1*	2.30±0.88	1–4	2.77±1.22	1–5	0.47±0.63	0–2	30
*shoc1*	2.38±1.04	0–5	2.79±1.41	0–7	0.41±0.61	0–2	34
*mus81*	5±0	5–5					30
*asy1/shoc1*	0.69±0.69	0–2	0.78±0.91	0–3	0.09±0.30	0–1	32
*asy1/mus81*	2.14±1.01	1–4	2.18±1.09	1–5	0.04±0.19	0–1	28

Average, SD, and range of bivalents, chiasmata, and ring bivalents per cell in Arabidopsis *asy1*, *shoc1*, *mus81* single mutants and *asy1/shoc1* and *asy1/mus81* double mutants. Note: the bivalent frequency between *asy1* and *asy1/mus81* was not significantly different, while the number of ring bivalents in *asy1/mus81* was reduced, causing the observed reduction in chiasma frequency. *n*=number of cells.

### Axis remodelling is defective in *pch2*

**Fig. 7.**
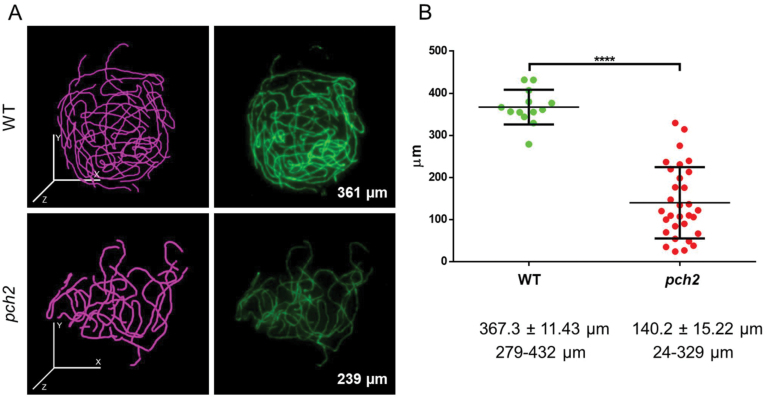
Compromised SC formation in *pch2*. (A) 3D reconstruction of the SC with simple neurite tracer (ImageJ) (pink, left) of representative pachytene cells immunolabelled with ZYP1 (green, right) in the WT and *pch2*. SC length is indicated in the bottom right corner. (B) SC measurements in individual WT and *pch2* cells. Average, SD, and range are indicated.

In *pch2*, ASY1 loaded onto chromosomes and, once ASY1 was fully polymerized, ZYP1 appeared initially as foci and later on as stretches. However, full ZYP1 polymerization was never observed (Fig. 3). Indeed, measuring ZYP1 extension in WT and *pch2-9* nuclei revealed a large variation in the extension of the SC across *pch2* cells and, on average, a reduction of 62% in SC length (Mann–Whitney–Wilcoxon test, *P*<0.0001, *n*=13 WT and *n*=31 *pch2*) (Fig. 7).

Furthermore, in contrast to the WT, in *pch2* ASY1 remained brightly stained following ZYP1 installation (Fig. 3), suggesting that ASY1 is not depleted from chromosome axes at synapsed regions. In various organisms including Arabidopsis, PCH2 is needed for the programmed removal of ASY1 during zygotene from chromosome axes concomitant with SC installation (Börner *et al.*, 2008; Martinez-Perez *et al.*, 2008; Wojtasz *et al.*, 2009; Lambing *et al.*, 2015). To validate this observation, we measured ASY1 intensity (Supplementary Table S4) and found that in the WT there was a 55% reduction in ASY1 intensity in synapsed versus asynapsed regions, whereas in *pch2* there was a 21% increase. This increase in *pch2* is probably based on the juxtaposition of the two homologous chromosome axes, leading to an increase in signal intensity per pixel.

Interestingly, in asynapsed regions, ASY1 intensity was consistently lower in *pch2* than in the WT. In Arabidopsis, PCH2 is involved not only in ASY1 removal, but also in its loading (Yang *et al.*, 2020b). To check whether ASY1 loading is also defective in *pch2*, we measured ASY1 intensity in leptotene whole nuclei. Despite substantial variation in ASY1 intensity values among slides/experiments, ASY1 intensity was consistently lower in *pch2* than in the WT (range 14–76%). To corroborate this cytological observation, we also immunostained ASY1 in Arabidopsis WT and *pch2*, and found that ASY1 signal intensity was significantly reduced at leptotene in Arabidopsis *pch2* (Mann–Whitney–Wilcoxon test, *P*=4.01^e-03^, *n*=24 Col-0 and *n*=24 *pch2*) (Supplementary Fig. S3). In summary, our data indicate that PCH2 in *B. rapa* is important for initial ASY1 loading during leptotene and programmed removal of ASY1 from chromosome axes concomitant with SC installation during zygotene.

### Crossover patterning in *pch2*: distalized crossover at the expense of interstitial crossover

In *pch2*, chiasma frequency was highly variable and in ~50% of cells the obligatory CO was lost. To better understand how CO control is altered, we analysed CO frequency distribution. We found that chiasma frequency in *pch2* did not significantly deviate from a Poisson-predicted distribution [χ ^2^_(*pch2-9*)_=14.70, *P*=0.26, *n*=98)] (Fig. 5), revealing a random distribution of chiasmata between cells. To identify whether interference-sensitive class I COs form in *pch2*, we performed immunolocalization of HEI10 together with ZYP1 (Fig. 6B). In *pch2-9*, we found an average of 10.5±3.15 (range 4–15) HEI10 foci during diplotene–diakinesis. These values are comparable with the average chiasma number in *pch2-9* (9.97±3.29, range 3–17). Interestingly, we found nuclei where several HEI10 foci localized in close proximity onto one ZYP1 stretch (Fig. 6B, asterisks) together with ZYP1 stretches devoid of HEI10 foci. Thus, a majority of COs in *pch2* showing a random distribution according a Poisson-predicted distribution probably belong to class I COs (marked by HEI10 foci).

**Fig. 8.**
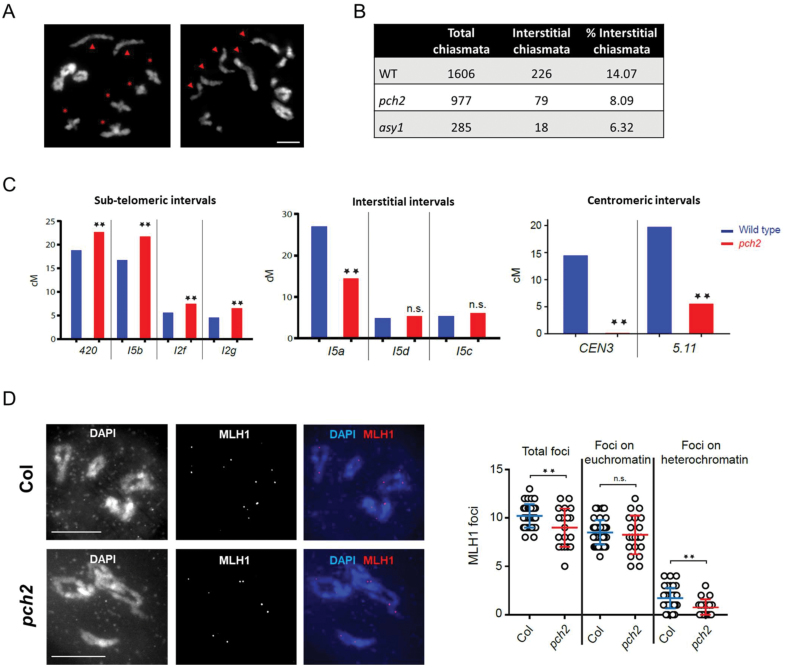
Distalization of chiasmata in *pch2* and in *asy1*. (A) Representative example of *B. rapa* diakinesis with a majority of chiasmata being interstitial (asterisks) in the WT (left) and distal (arrowheads) in *pch2* (right). DNA is counterstained with DAPI and shown in grey. Scale bar=5 µm. (B) Quantification of interstitial versus total chiasmata in *B. rapa* WT, *pch2*, and *asy1* reveals reduced interstitial CO frequency in the mutants. (C) Recombination frequency in Arabidopsis at genetic intervals defined with genes coding for fluorescent proteins. Genetic intervals are classed based on their locations on the chromosomes. Asterisks represent statistically significant differences (generalized linear model) and ‘n.s.’ means non-significant. (D) Left: staining of MLH1 (red) and DAPI (blue) at diakinesis in Arabidopsis WT and *pch2*. Scale bars=10 µM. Right: plots showing total MLH1 foci count, MLH1 foci count on euchromatin, and foci count on heterochromatin per cell. Error bars represent the SD. Asterisks represent statistically significant differences (Mann–Whitney–Wilcoxon test) and ‘n.s.’ means non-significant.

We next asked whether residual chiasmata in *pch2* are positioned differently compared with the WT. We scored the number of terminal versus interstitial chiasmata and found that the percentage of interstitial chiasmata among all chiasmata was 8% in *pch2* and 14% in the WT (Fig. 8A, B). Similarly, in *asy1*, among residual chiasmata, only 6% were interstitial while the majority were found towards chromosome ends (Fig. 8B). Thus, cytologically residual chiasmata in *pch2* and *asy1* are proportionally more frequently located in terminal chromosome regions compared with the WT.

To corroborate our cytological finding on altered chiasma patterning in *pch2* with proportionally increased terminal and decreased interstitial chiasmata, we asked whether in Arabidopsis *pch2* a similar situation occurs. We re-analysed published recombination data from six genetic intervals (Lambing *et al.*, 2015) and extended this study by analysing recombination frequency in three more genetic intervals. We classed the genetic intervals based on their chromosomal locations as subtelomeric, interstitial, and centromeric. We found that subtelomeric intervals show increased recombination frequency, while interstitial intervals show few changes, and centromeric intervals show a drastic reduction in recombination frequency in *pch2* (Fig. 8C). These data suggest that COs are repressed in the heterochromatic centromeric regions and that they are distalized in the absence of PCH2. Immunostaining of MLH1 revealed that total MLH1 foci are reduced in *pch2* (10.4 versus 9.0, Mann–Whitney–Wilcoxon test, *P*<0.01, *n*=41 WT and *n*=20 *pch2*) and that the reduction is mostly seen on the DAPI-dense regions that are representative of the heterochromatic (peri-)centromeric regions (1.7 versus 0.8, Mann–Whitney–Wilcoxon test, *P*<0.001, *n*=41 WT and *n*=20 *pch2*) (Fig. 8D). Together, these data reveal that PCH2 is required for CO patterning control.

## Discussion

In this work, we have characterized *asy1* and *pch2* in *B. rapa*. Both mutants display reduced chiasma formation and univalents at metaphase I that lead to unbalanced gametes and reduced fertility, highlighting the importance of the axis and SC morphogenesis for CO formation. Depletion of ASY1 or PCH2 strongly impacts SC formation, resulting in differential patterning of residual chiasmata being proportionally enriched towards chromosome ends.

### ASY1-independent meiotic chromosome axes are insufficient for synaptonemal complex and crossover formation

Our analyses indicate that *asy1-13* and *asy1-14* are allelic and probably represent null mutations. Similar to *asy1* in other plant species (Armstrong *et al.*, 2002; Nonomura *et al.*, 2004), *B. rapa asy1* is largely, if not completely, asynaptic (Figs 1D, 3). ZYP1 forms foci and occasionally short stretches which are commonly distorted in appearance (Fig. 3), probably representing ZYP1 aggregates (Zickler and Kleckner, 1999) or polycomplexes forming at sites where ZYP1 polymerization/extension along lateral elements is impaired due to a defect in ASY1-dependent interhomologue bias and recombination progression (Higgins *et al.*, 2005).

Despite the absence of an SC, residual chiasmata form in *B. rapa asy1* (Fig. 1C, D). This is reminiscent of Arabidopsis and the corresponding *hop1* mutant in budding yeast (Hollingsworth and Johnson, 1993; Ross *et al.*, 1997) but contrasts with rice *pair2* (Nonomura *et al.*, 2004). Axis mutants *asy3* or *asy4* in Arabidopsis also show reduced COs (but to a lesser extent than *asy1*), and *asy1* is epistatic to them in terms of chiasma frequency (Ferdous *et al.*, 2012; Chambon *et al.*, 2018), suggesting that ASY1 is needed upstream from ASY3/ASY4 to regulate DMC1 dynamics and thus interhomologue recombination (Sanchez-Moran *et al.*, 2007). Moreover, ASY4 is required for axial organization of ASY1 and ASY3 in Arabidopsis (Chambon *et al.*, 2018), and ASY3 for correct ASY1 localization in Arabidopsis, maize, and rice (Wang *et al.*, 2011; Ferdous *et al.*, 2012; Lee *et al.*, 2015), while cytologically ASY3 and the cohesin SMC3 localize correctly in *asy1* (Sanchez-Moran *et al.*, 2007; Ferdous *et al.*, 2012). Similarly, in *B. rapa*, SMC3 and ASY3 display WT-like axes localization in *asy1* (Fig. 4), suggesting WT-like dynamics of the core axis-associated proteins despite subsequent synaptic and CO defects.

In Arabidopsis axis mutants, the majority of the remaining COs are dependent on the class I CO (ZMM) pathway similar to the WT; for example, in *asy1*, MLH1 foci form on bivalents (Lambing *et al.*, 2020), in *asy3*/*msh4* double mutants chiasma formation is abolished (Ferdous *et al.*, 2012), or in *asy4*/*zip4* and *asy4*/*msh5* double mutants bivalent formation is reduced by ~95% compared with *asy4* (Chambon *et al.*, 2018). In *B. rapa asy1*, both HEI10 and MLH1 foci numbers scored around pachytene-/diplotene-like stages were highly variable compared with the WT, exceeding the number of cytologically scored chiasmata. This observation could be attributed to different causes. First, HEI10 foci numbers vary significantly through prophase I and therefore variation could stem from the difficulty in selecting late prophase cells due to complete absence of synapsis. Second, interestingly, in Arabidopsis *asy1*, *dmc1* or haploid meiosis MLH1 foci were reported on univalent chromosomes, possibly representing sites of intersister repair (Cifuentes *et al.*, 2013; Lambing *et al.*, 2020). Similarly, HEI10 has been indicated to mark intersister events (Voelkel-Meiman *et al.*, 2012) and it follows initial WT dynamics in mutants with severely impaired CO formation such as the ZMM mutant *mer3* (Wang *et al.*, 2012). Third, non-co-localizing HEI10 and MLH1 foci were found in *B. napus* allohaploids (Grandont *et al.* 2014). Finally, MLH1 in *Sordaria* is important for interlock resolution (Storlazzi *et al.*, 2010), and although the presence of MLH1 at interlocks has not been shown, this could arise in *asy1* due to defective SC polymerization. Assuming that only ZYP1-associated ZMM foci or a subset of these are CO competent (Ferdous *et al.*, 2012), then probably only a proportion of observed foci will mature into class I CO sites.

In *B. rapa asy1*, chiasmata were strongly reduced but not abolished, and cytologically foci corresponding to class I CO markers HEI10 and MLH1 formed, suggesting that residual chiasmata may arise via the class I CO pathway. In support of this, by using available Arabidopsis resources, we showed that a majority of ASY1-independent chiasmata are dependent on the ZMM pathway, while a subset is also MUS81 dependent, indicative of class II CO formation (Table 1). Interestingly, as in the WT, roughly similar proportions of class I and class II COs arise in the Arabidopsis *asy1* mutant, suggesting that both CO pathways are similarly dependent on ASY1. In future, to determine if the same applies in *B. rapa*, it will be interesting to evaluate CO formation in *asy1/msh4* (class I CO-defective mutant with ~4 residual chiasmata; Blary *et al.*, 2018).

### Residual chiasmata in *asy1* are proportionally enriched in terminal chromosome regions and on the major 45S rDNA-carrying chromosome

Residual chiasmata in *B. rapa asy1* are proportionally enriched towards chromosome ends and, except for the major 45S rDNA-carrying chromosome, all discernible chromosomes appear as univalents with similar frequencies, suggesting no positive correlation between chromosome size and chiasma formation.

Terminal dominance of residual chiasmata was also found in Arabidopsis *asy1* and further axis-associated mutants such as *asy3* and *asy4* (Sanchez-Moran *et al.*, 2007; Ferdous *et al.*, 2012; Chambon *et al.*, 2018). It has been proposed that this could be a consequence of recombination initiating by telomeric regions (Sanchez-Moran *et al.*, 2007) and that ASY1 could be antagonizing this telomere-led recombination (Lambing *et al.*, 2020). This highlights the key role of axis components in regulating CO frequency and distribution.

Notably, residual chiasmata showed a strong bias towards the 45S rDNA-carrying chromosome and, in particular, towards the 45S rDNA chromosome end (Fig. 2). Due to limited cytological resolution, we cannot distinguish whether chiasmata involved ribosomal repeats or rather formed in non-ribosomal repeat-free regions distal to the 45S rDNA or intermingled with it. We prefer that CO enrichment is found in non-ribosomal repeat-free chromosome ends, as bivalents were typically linked via what seems to be cytologically a very terminal chiasma, and also because we could not see any signs of repair/chromosome segregation problems which would be likely to arise if recombination would occur within highly repetitive regions due to the possibility of non-allelic exchanges (Sasaki *et al.*, 2010). This would agree with recent data from Arabidopsis indicating that exclusion of ASY1 from the NOR in chromosomes 2 and 4 early during prophase is implicated in restricting DSB formation and thus HR in the NOR (Sims *et al.*, 2019). Nonetheless, chiasma enrichment towards the 45S rDNA-carrying chromosome (arm) has also been observed in Arabidopsis *asy1* (Sanchez Moran *et al*., 2001; Lambing *et al.*, 2020). Thus, in *asy1*, the rDNA probably promotes CO formation in the 45S rDNA-carrying chromosome (arm) (Sanchez Moran *et al*., 2001; Lambing *et al.*, 2020).

### *pch2* plants fail to remodel ASY1 and only achieve partial synapsis leading to reduced chiasma formation

Both *pch2-9* and *pch2-12* appear allelic in terms of meiotic behaviour and probably represent null mutants. However, despite three backcrosses, compared with the WT, a slight reduction in fertility was found in *pch2-12* segregating families independent of *PCH2*, suggesting secondary mutation load impacting overall plant fertility. Thus, data were acquired on *pch2-9*.

In *B. rapa pch2*, full synapsis was never observed (Figs 1D, 3) despite substantial variation in SC extension (based on ZYP1 immunolocalization) and, on average, SC length was ~62% reduced compared with the WT (Fig. 7). This reduction in SC length is similar to *pch2* in Arabidopsis (~68%; Lambing *et al.*, 2015) but differs from rice *pch2/crc1*, where SC formation is completely abolished (Miao *et al.*, 2013). Notably, in rice *pch2*, no chiasmata form, while in *B. rapa pch2* chiasmata are reduced by ~40% and in Arabidopsis *pch2* by ~30%. Moreover, observed chiasma numbers are variable across cells, and often univalents occurred in cells with at least 10 chiasmata, suggesting a defect in CO assurance. Overall, despite species-specific differences, PCH2 in plants is critical for WT levels of synapsis and CO formation.

*Brassica rapa pch2* plants form a partial SC while axes are not remodelled; that is, ASY1 loading during leptotene is reduced and ASY1 is not depleted from synapsed regions. This dual function of PCH2 in *B. rapa* is consistent with data from Arabidopsis and budding yeast (Börner *et al.*, 2008; Lambing *et al.*, 2015; Yang *et al*., 2020a, b), showing that PCH2 is critical for chromosomal localization of ASY1 as well as for depletion of ASY1 from synapsed regions. In Arabidopsis, in addition to PCH2, the axis proteins ASY3 and ASY4 are also critical for WT dynamics and correct localization of ASY1; for example, in *asy4*, ASY1 is not depleted from synapsed regions and ASY3 is involved in correct ASY1 recruitment to the chromosome axes (Ferdous *et al.*, 2012; Chambon *et al.*, 2018). Thus, a complex interplay between axis components and associated proteins exists, and not only axis formation but also axis morphogenesis is critical for HR, including DSB repair, template choice, SC formation and, ultimately, CO formation.

### Redistribution of chiasmata in *pch2*

Residual COs in *B. rapa pch2*, contrary to Arabidopsis (Lambing *et al.*, 2015), do not significantly deviate from a Poisson-predicted distribution which could be interpreted as suggesting that they are interference insensitive, typical for class II COs. However, the fact that the chiasma frequency distribution does not differ from a Poisson distribution only means that chiasma formation in the mutant has a high random component. In fact, HEI10 foci marking putative class I CO sites appear in *pch2* with similar numbers as cytologically scored chiasmata (minimum CO number), with some nuclei having HEI10 foci closely spaced along ZYP1 stretches and at the same time ZYP1 stretches devoid of HEI10 foci (Fig. 6). Possibly maturation of designated COs might be compromised in *B. rapa pch2* due to the defect in chromosome axis remodelling, resulting in a CO deficit as in Arabidopsis *pch2* (Lambing *et al.*, 2015). Notably, the average reduction in SC length (62%) is larger than the average reduction in chiasmata (~40%), suggesting that similar to Arabidopsis (Lambing *et al.*, 2015), the mean reduction in CO frequency was not coordinated with that in SC length. A similar phenotype has been observed in Arabidopsis mutants for the kinesin PSS1 and the E1 enzyme of the neddylation complex AXR1 (Duroc *et al.*, 2014; Jahns *et al.*, 2014), both being strongly defective for synapsis, showing univalents at metaphase I, and with chiasma frequencies similar to the WT but COs being redistributed with closely spaced HEI10 foci along residual synapsed regions.

Furthermore, PCH2-independent chiasmata are cytologically skewed towards terminal chromosome regions in *B. rapa*. To support this cytological observation, in Arabidopsis *pch2*, CO rates were established in different genetic intervals, revealing increased CO rates in telomeric chromosome regions at the expense of decreased (peri-)centromeric CO rates compared with the WT (Fig. 8). Thus, considering terminal initiation of SC formation (Hurel *et al.*, 2018), recombination events on limited extended ZYP1 stretches in *pch2* might be proportionally enriched in distal regions, explaining the skewed chiasma distribution.

Altogether, in *B. rapa*, a majority of residual PCH2-independent COs are likely to be ZMM-dependent class I COs that are redistributed, being proportionally enriched towards chromosome ends.

### Conclusions

Altering CO frequency and distribution is of special interest for plant breeding, since in many crop species CO numbers are limited and skewed towards chromosome ends, limiting recombination and access to naturally available genetic variation and creating linkage drag.

We have analysed mutants of axis-associated ASY1 and axis-remodelling PCH2. Both mutants show reduced CO formation due to defects in meiosis, but interestingly COs are redistributed and at least *pch2* plants produce some viable seeds. Considering this altered patterning of chiasmata that arise via the class I and class II CO pathways in *asy1* and *pch2*, it would be interesting to determine whether in combination with HEI10 overexpression (increasing class I CO) or *hyperrec* mutants (increasing class II COs) (Lambing *et al.*, 2017). ASY1-/PCH2-independent chiasmata could be increased, thus potentially increasing bivalent formation and seed setting while maintaining an altered recombination pattern. In any case, considering their general influence on DSB and CO formation including DNA repair template choice, axis-associated or remodelling proteins such as ASY1 or PCH2 are interesting targets to modify meiotic recombination landscapes in the context of plant breeding.

## Supplementary data

The following supplementary data are available at *JXB* online.

Table S1. Primers used in this study.

Table S2. Plant fertility in the *B. rapa* WT, and *asy1* and *pch2* mutants.

Table S3. Relative occurrence (%) of 5S/45S-labelled chromosomes as either univalents or bivalents.

Table S4. ASY1 immunofluorescence relative signal intensity in WT and *pch2* cells.

Fig. S1. Alternative splicing of *PCH2* in *pch2-12*.

Fig. S2. Male meiotic chromosome behaviour in *B. rapa.*

Fig. S3. Localization defect of ASY1 in Arabidopsis *pch2*.

erab035_suppl_Supplementary_Tables_S1-S4_and_Figures_S1-S3

## Data Availability

All data supporting the findings of this study are available within the paper and within its supplementary data published online.

## Abbreviations

CO: crossover
DSB: DNA double-strand break
FISH: fluorescence *in situ* hybridization
HR: homologous recombination
NCO: non-crossover
SC: synaptonemal complex
WT: wild type
