# Supplementary material for: Meiotic chromosome axis remodelling is critical for meiotic recombination in Brassica rapa
Source: J Exp Bot. 2021 Jan 27;72(8):3012–27. doi: 10.1093/jxb/erab035 (PMC8023211; doi:10.1093/jxb/erab035)
Supplement: erab035_suppl_Supplementary_Tables_S1-S4_and_Figures_S1-S3 [file erab035_suppl_supplementary_tables_s1-s4_and_figures_s1-s3.pdf]

Supplementary Table S1. Primers used in this study. For dCAPS genotyping, restriction enzymes are indicated as well as corresponding fragment sizes after digestion. Highlighted nucleotides are non-complementary bases introduced to generate corresponding restriction sites.

Gene	Primer name	Sequence 5' to 3'	Restriction enzyme	Size restriction fragments (bp)	
				WT	Mutant
**dCAPS genotyping**					
PCH2-9	PGF2	TGTAGATGGCCTTGTTCTCATACCTC	XbaI	224	196+28
	P9R	GGCATGGACAACAGGCTTAACTT**T**C			
PCH2-12	PGF2	TGTAGATGGCCTTGTTCTCATACCTC	EcoRV	260	232+28
	P12R	CAGTGCCTTATTCAACTTTTGCATA**G**ATA			
ASY1-13	A13F	GAACTTTTAACTCCACTGCTGACATTA**AT**	PacI	123	94+29
	A13R	GCACAATCACGAAAATTACAAAGCATG			
ASY1-14	A14F	CGGTTCCTGCTCTAGGTAACAAGC	EcoRI	185	148+37
	A14R	GCTAAAATCATTAGAATACAAAAAAACCTTTCT**GA**AT			
**Reverse transcription-PCR**					
Actin	Actin_F	GAGCAACTGGGATGACATGG			
	Actin_R	GAAACCCTCGTAGATTGGCAC			
PCH2-12	PCH2_RT_F1	CTCGTCTCTGTGGAGGTCTG			
	PCH2_RT_R2	GACCCAGTTCCTCACACG			
**T-DNA genotyping**					
asy1-4	ASY1_F	TCAGCTGGAGTTTTTAGCTCG			
	ASY1_R	GAACCATTTTGCAAGCTGAAC			
shoc1-1	SHOC1_F	AGCTTAAGCGAATCTTACCGG			
	SHOC1_R	TGGTTCAATGGCTTTTTGAAG			
mus81-2	MUS81_F	TGGTGAAATCTAGCAACCCAG			
	MUS81_R	AATTTTCCACAAACCCTTTGG			
pch2-1	PCH2_F	CAGTGCAAATAGCCGTCGCTGAG			
	PCH2_R	CTCACATGGTCCTTCTTCAATGAGC			
T-DNA	LB_salk_LB1.3	ATTTTGCCGATTTCGGAAC			

Supplementary Table S2. Plant fertility in *B. rapa* WT, *asy1* and *pch2* mutants. Silique length and seed setting in *B. rapa* WT and in TILLING plants WT, heterozygous or mutant for *ASY1* and *PCH2* including allelic mutants. N = total number of siliques (10 siliques per plant).

	Average silique length (cm)	Average seed set	N
WT	7.60 ± 0.61	37.73 ± 6.73	120
ASY1-13 +/+	7.10 ± 0.60	37.84 ± 4.94	50
ASY1-13 +/-	7.48 ± 0.51	36.02 ± 5.63	50
asy1-13	2.80 ± 0.34	0 ± 0	50
ASY1-14 +/+	7.49 ± 0.55	37.32 ± 5.52	50
ASY1-14 +/-	7.86 ± 0.65	38.7 ± 6.58	50
asy1-14	3.05 ± 0.67	0 ± 0	50
asy1-13/14	3.16 ± 0.20	0 ± 0	50
PCH2-9 +/+	7.40 ± 0.50	36.38 ± 4.22	50
PCH2-9 +/-	7.17 ± 0.41	34.16 ± 5.39	50
pch2-9	4.41 ± 1.01	2.74 ± 3.60	50
PCH2-12 +/+	6.80 ± 0.56	35.32 ± 8.09	50
PCH2-12 +/-	6.56 ± 0.54	33.20 ± 6.34	50
pch2-12	3.19 ± 0.79	1.02 ± 2.32	50
pch2-9/12	4.28 ± 1.08	3.22 ± 1.01	50

Supplementary Table S3. Relative occurrence (%) of 5S/45S-labelled chromosomes as either univalents or bivalents. Chromosome #3 formed a bivalent with significantly higher frequency than all other discernible chromosomes. N = 73 *asy1* diakinesis cells.

	#1 (5S)	#3 (5S+45S)	#4 (5S+45S)	#5 (5S+45S)	#6 (45S)	#10 (5S)
Univalent	84.93	36.76	79.45	84.93	86.30	82.19
Bivalent	15.07	63.24	20.55	15.07	13.70	17.81

Supplementary Table S4. ASY1 immunofluorescence relative signal intensity in WT and *pch2* cells. A. Asynapsed vs synapsed regions. In *pch2*, ASY1 is not depleted from synapsed regions and remains brightly stained in pachytene cells. **B**. Whole leptotene nuclei. Initial ASY1 loading is impaired in *pch2*. Independent experiments performed with two independent microscopes. Values represent nuclei fluorescence intensity after deduction of background signal intensity (see methods). N = number of cells. Asterisks indicate significant differences (Student's *t* test, P < 0.05).

A

WT			*pch-2*		
Asynapsed	Synapsed	Difference	Asynapsed	Synapsed	Difference
1922.41<br>N=11	846.38<br>N=10	-55% (*)	1758.09<br>N=16	2137.14<br>N=17	+21% (*)

B

Whole nuclei	WT	*pch2*	Difference
Microscope 1	447.09<br>N=21	348.79<br>N=31<br>346.84<br>N=8	-22% (*)<br><br>-22%
Microscope 1	1392.20<br>N=23	331.07<br>N=23	-76% (*)
Microscope 2	3943.67<br>N=43	3388.21<br>N=21	-14%

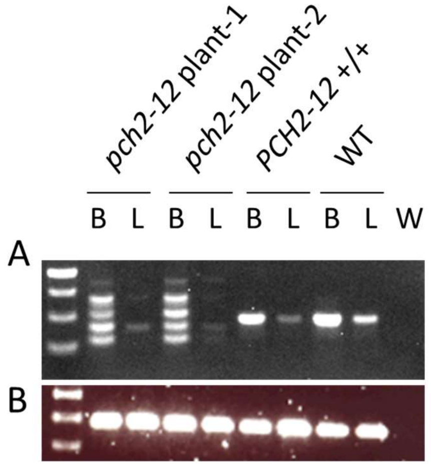

Supplementary Fig. S1. Alternative splicing of *PCH2* in *pch2-12*. Reverse transcription-PCR for *PCH2* in two independent mutant plants, one sibling TILLING plant WT for *PCH2* and one WT plant. **A**. Primer pair spanning the mutation site. Note alternative splicing variants in mutant plants only. **B.** Expression of actin as technical control. B: bud; L: leaf; W: water control.

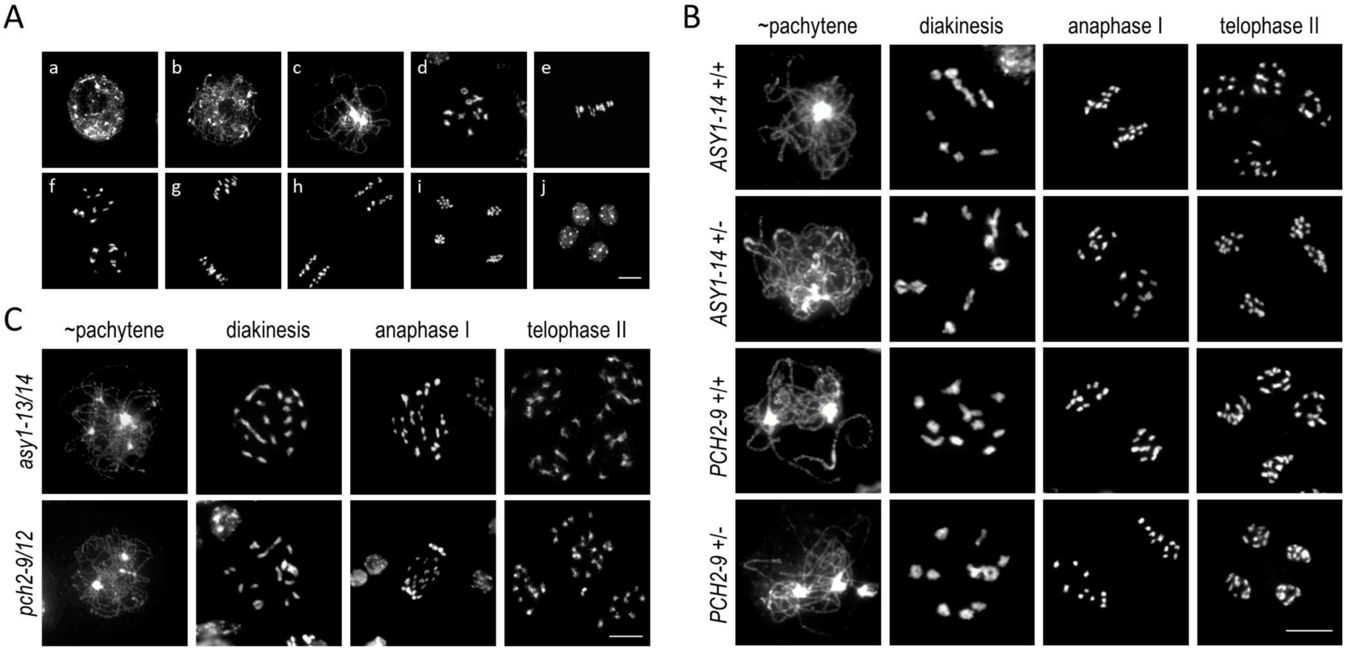

Supplementary Fig. S2. Male meiotic chromosome behaviour in *B. rapa*. A. Meiotic stages in *B. rapa* WT. **a**. Leptotene. **b**. Zygotene. **c**. Pachytene. **d**. Diakinesis. **e**. Metaphase I. **f**. Telophase I. **g**. Metaphase II. **h**. Anaphase II. **i**. Telophase II. **j**. Tetrad. **B**. WT meiosis in TILLING plants WT or heterozygous for *ASY1* and *PCH2*. **C**. Meiosis in the allelic mutants *asy1-13/14* and *pch2-9/12*. DNA counterstained with DAPI and shown in grey. Bars = 10µm.

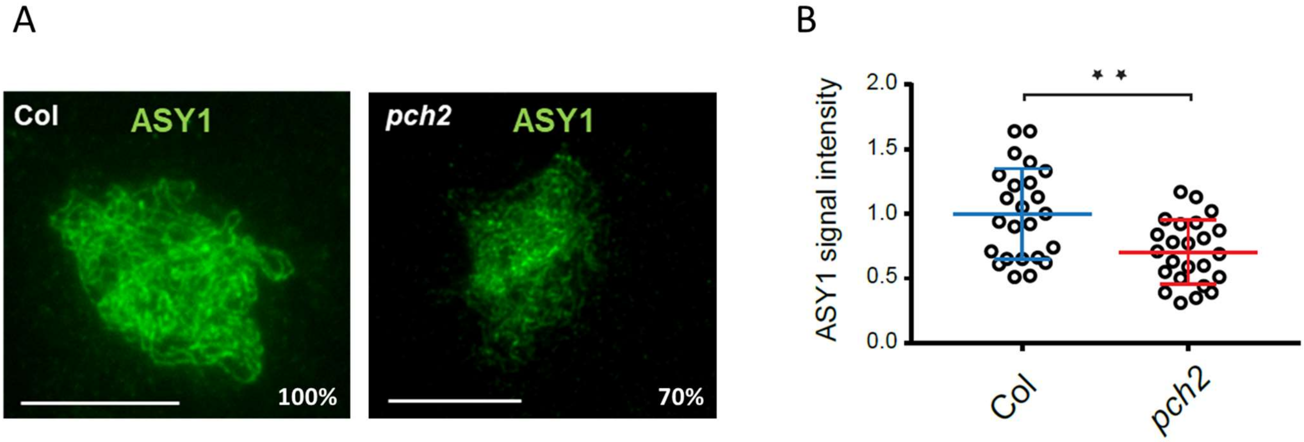

Supplementary Fig. S3. Localization defect of ASY1 in Arabidopsis *pch2*. A. Staining of ASY1 at leptotene in WT and *pch2*. ASY1 relative signal intensity indicated in the bottom right corner. Bars = 10μm. **B**. Plot showing relative ASY1 signal intensity in each background. Asterisks represent statistically significant differences (MWW test).
